# Current surgical treatment strategies and ongoing issues for locally recurrent rectal cancer

**DOI:** 10.1093/jjco/hyaf127

**Published:** 2025-08-07

**Authors:** Yasuyuki Yokoyama, Kay Uehara, Takeshi Yamada, Aitsariya Monkhonsupphawan, Seiichi Shinji, Akihisa Matsuda, Goro Takahashi, Woramin Riansuwan, Hiroshi Yoshida

**Affiliations:** Department of Gastroenterological Surgery, Nippon Medical School, 1-1-5 Sendagi, Bunkyo-ku, Tokyo 113-8602, Japan; Department of Gastroenterological Surgery, Nippon Medical School, 1-1-5 Sendagi, Bunkyo-ku, Tokyo 113-8602, Japan; Department of Gastroenterological Surgery, Nippon Medical School, 1-1-5 Sendagi, Bunkyo-ku, Tokyo 113-8602, Japan; Department of Gastroenterological Surgery, Nippon Medical School, 1-1-5 Sendagi, Bunkyo-ku, Tokyo 113-8602, Japan; Department of Surgery, Siriraj Hospital, Mahidol University, Wanglang Road Bangkoknoi, Bangkok 10700, Thailand; Department of Gastroenterological Surgery, Nippon Medical School, 1-1-5 Sendagi, Bunkyo-ku, Tokyo 113-8602, Japan; Department of Gastroenterological Surgery, Nippon Medical School, 1-1-5 Sendagi, Bunkyo-ku, Tokyo 113-8602, Japan; Department of Gastroenterological Surgery, Nippon Medical School, 1-1-5 Sendagi, Bunkyo-ku, Tokyo 113-8602, Japan; Department of Surgery, Siriraj Hospital, Mahidol University, Wanglang Road Bangkoknoi, Bangkok 10700, Thailand; Department of Gastroenterological Surgery, Nippon Medical School, 1-1-5 Sendagi, Bunkyo-ku, Tokyo 113-8602, Japan

**Keywords:** locally recurrent rectal cancer, R0 resection, pelvic exenteration, minimally invasive surgery, carbon ion radiotherapy

## Abstract

Locally recurrent rectal cancer (LRRC) remains one of the most challenging problems in the rectal cancer management, despite advances in multimodal treatments. R0 resection remains the cornerstone of curative therapy and the most critical prognostic factor. However, achieving R0 resection is technically demanding, with outcomes heavily influenced by tumor location, institutional expertise, and careful patient selection. This narrative review summarizes current surgical strategies for LRRC, emphasizing the importance of accurate anatomical classification, multidisciplinary collaboration, and individualized planning. Extended resections—including bony pelvis, pelvic sidewall, and vascular dissections—have expanded surgical indications but require specialized expertise and carry risks of functional impairment. Minimally invasive approaches, such as laparoscopic or robotic pelvic exenteration, may offer potential advantages in selected cases but remain technically challenging. Carbon ion radiotherapy, which demonstrates superior local control compared to conventional radiotherapy, is expected to be a promising treatment for unresectable LRRCs. Its future role as an alternative or perioperative treatment for resectable or borderline cases is under investigation. Preoperative chemoradiotherapy may play an important role in radiation-naïve patients, while re-irradiation strategies remain controversial for previously irradiated cases. In patients with resectable distant metastases, aggressive combined surgical approaches may be pursued if curative resection is feasible. Ultimately, shared decision-making with patients is essential for optimal management of LRRC, based on a highly individualized, evidence-based approach that balances oncological prognosis and postoperative quality of life.

## Teaser

Treatment for locally recurrent rectal cancer requires an individualized approach that carefully balances surgical risk with long-term oncological and functional outcomes, with the goal of achieving R0 resection.

## Introduction

Locally recurrent rectal cancer (LRRC) is typically defined as the reappearance of a tumor in the true pelvis following previous radical treatment for rectal cancer, involving lateral pelvic lymph node (LPLN) recurrence [1, 2]. In contrast, peritoneal dissemination that extends beyond the pelvic cavity is generally excluded from the definition of LRRC. However, peritoneal recurrence confined to the pelvic cavity—such as dissemination limited to the surface of the TME plane—is typically included as LRRC. Similarly, local recurrence after endoscopic resection is usually considered separately from LRRC. Additionally, tumor regrowth following the “watch-and-wait” strategy for clinical complete responder is not categorized as LRRC.

LRRC remains a complex clinical challenge despite advances in surgical techniques and multidisciplinary treatment. Over the years, advances in surgical techniques as well as the combination of radiotherapy and systemic treatment have led to improvements in treatment outcomes in selected cases. However, questions remain about how to select candidates for surgery, how to define the extent of resection, how to combine preoperative treatment and intraoperative radiotherapy, and the indications for new treatment methods such as carbon ion radiotherapy (CIRT).

This review provides a comprehensive narrative of the literature on LRRC management, with a particular focus on current surgical-based treatment strategies and ongoing issues. A comprehensive search of the literature written in English was performed up to March 2025, using the PubMed, Embase, and Cochrane databases. Search terms included “rectal cancer,” “locally recurrent,” “local recurrence,” and “local relapse”. While surgical intervention remains a cornerstone of LRRC management, the detailed technical aspects of these procedures are beyond the scope of this manuscript. For an in-depth discussion of surgical techniques, readers are encouraged to consult specialized surgical literature.

## Risk factors for local recurrence after rectal cancer resection

LRRC primarily arises from residual tumor cells at the distal rectal margin, tumor dissemination along the mesorectal excision surface, or undetected LPLN metastasis. The risk factors for LRRC can be broadly categorized into tumor-related factors, which reflect the biological behavior of the primary tumor, and surgeon-related factors, which are associated with the technical quality of surgery.

Among tumor-related factors, the presence of enlarged LPLNs or confirmed pathological metastases to these nodes is a strong predictor of local recurrence [3, 4]. Other high-risk pathological features include mesorectal lymph node metastasis, T4 tumors, greater tumor size, poorly differentiation, lymphovascular invasion, and perineural invasion are also strongly associated with local recurrence [5–8]. These characteristics reflect aggressive tumor biology and a higher propensity for local spread or incomplete clearance.

Surgical technique also plays a critical role in LRRC risk. Intraoperative mesorectal injury, a positive or close circumferential resection margin (CRM) of <1 mm, along with an inadequate distal resection margin, are all strongly associated with increased local recurrence. These parameters are often referred to as surgeon-related factors and are closely tied to surgical expertise, experience, and institutional volume. This issue is particularly relevant in the context of transanal total mesorectal excision (taTME), a technique developed to improve access to the narrow and deep pelvis in low rectal cancer surgery. An increased risk of local recurrence has been reported, particularly during the early phase of taTME adoption. A Dutch multicenter study reported a local recurrence rate of 10.0% in the first 120 taTME cases [9]. However, when the initial 10 cases at each institution were excluded, the recurrence rate dropped to 4.0%, demonstrating a steep learning curve. More recently, the TaLaR trial reported a 3-year local recurrence rate of 3.6% for taTME, comparable to 4.4% for laparoscopic TME, supporting the oncologic safety of taTME when performed by experienced surgeons [10].

Notably, local recurrence is not confined to advanced cases. A systematic review reported that ~25% of LRRC cases had a primary tumor with a depth of invasion classified as T1–T2, and that 48% had no lymph node metastasis [11]. This unexpectedly high recurrence rate among early-stage rectal cancers suggests that technical quality of surgery is a major contributing factor. Further evidence highlights the importance of subspecialty training and surgical volume. In one study, patients operated on by non–colorectal-trained surgeons had a 2.5-fold higher risk of local recurrence (*P* = .001), while those treated by surgeons with <21 annual rectal resections had a 1.8-fold increased risk (*P* < .001) [12]. Moreover, disease-specific survival was significantly worse among patients treated by non-specialists (hazard ratio (HR) = 1.5, *P* = .03) and those treated at low-volume centers (HR = 1.4, *P* = .005). These findings point to the critical need to optimize treatment in specialized centers through a combination of meticulous surgical technique and multimodal therapy to minimize local relapse and improve long-term outcomes.

## Actual reported local recurrence rate

The reported incidence of LRRC varies across studies, influenced by tumor stage, use of neoadjuvant therapy, surgical approach, and follow-up duration. Based on data from large randomized and observational studies conducted in Japan and internationally, the 3-year local recurrence rate after curative surgery for locally advanced rectal cancer ranges from 2.6% to 11%, while the 5-year rate ranges from 5.6% to 16.5%, depending on study design and patient characteristics (Table 1) [13–22].

**Table 1:** Summary of local recurrence rates after rectal cancer surgery from large clinical trials

Comparative study of preoperative treatment						
Trial	Author	Year	Preoperative treatment	Cases	Proportion of cStage III	Local recurrence rate
German trial	Sauer [13]	2004	RT	405	54%	6% (5-yr)
			Postoperative RT	394	51%	13% (5-yr)
FFCD 9203	Gerard [14]	2006	RT	367	38%	16.5% (5-yr)
			CRT	375	38%	8.1% (5-yr)
Dutch trial	Peeters [15]	2007	None	908	36%	10.9% (5-yr)
			SCRT	897	34%	5.6% (5-yr)
RAPIDO	Bahadoer [16]	2021	SCRT + Cap	462	91%	6.8% (3-yr)
			Cap + RT	450	92%	4.3% (3-yr)
PRODIGE 23	Conroy [17]	2021	FOLFIRINOX + CRT	231	90%	4% (3-yr)
			Cap + RT	230	90%	6% (3-yr)
STELLAR	Jin [18]	2022	SCRT + CAPOX	302	86%	8.4% (3-yr)
			Cap + RT	297	84%	11.0% (3-yr)
**Comparative study of surgical approach**						
Trial	Author	Year	Surgical approach	Cases	Proportion of cStage III	Local recurrence rate
CLASICC	Jayne [19]	2007	LAP	526	49%	7.8% (3-yr)
			Open	268	45%	7% (3-yr)
COREAN	Jeong [20]	2014	LAP	170	65%	2.6% (3-yr)
			Open	170	69%	4.9% (3-yr)
COLOR II	Bonjer [21]	2015	LAP	699	37%	5% (3-yr)
			Open	345	37%	5% (3-yr)
Japanese Cohort Study	Hida [22]	2018	LAP	574	57%	10.1% (3-yr)
			Open	926	65%	8.5% (3-yr)

RT, radiotherapy; CRT, chemoradiotherapy; SCRT, short-course radiotherapy; Cap, capecitabine; LAP, laparoscopic.

In addition, the Japanese randomized controlled trial JCOG0212, which compared total mesorectal excision (TME) alone with TME plus lateral lymph node dissection, reported a 5-year local recurrence rate of 14% and 7%, respectively, demonstrating the impact of surgical technique on local control [23].

In Japan, a 2007 report from the national registry of the Japanese Society for Cancer of the Colon and Rectum found a local recurrence rate of 4.1% (excluding anastomotic recurrences), reflecting relatively favorable oncological outcomes at leading centers [24]. However, these data predominantly represent institutions with advanced surgical expertise and may not capture the broader national landscape, where treatment is delivered across a wide range of facilities with varying levels of expertise. To better reflect real-world outcomes, a large-scale retrospective multicenter observational study conducted by the Japan Society of Laparoscopic Colorectal Surgery evaluated patients treated between 2010 and 2011. This study reported local recurrence rates of 8.5% following open surgery and 10.1% following laparoscopic surgery for advanced lower rectal cancer, indicating the persistent risk of LRRC even in the era of minimally invasive techniques [22]. Based on these and other observational data, the nationwide local recurrence rate for lower rectal cancer in Japan is estimated to fall between 10% and 15%.

Internationally, local recurrence rate reported in randomized controlled trials (RCTs) have declined over the past two decades, largely due to the introduction of preoperative chemoradiotherapy (CRT). In the 2000s, the 5-year local recurrence rate after preoperative radiotherapy (RT) or CRT followed by TME for stage II/III rectal cancer was reported to be ~5%–10% [13–15]. In more recent RCTs comparing open and minimally invasive surgery, the 3-year local recurrence rate has been reported to be 2%–7% [19–21]. However, in these trials, the proportion of patients with stage III disease was generally ~50%. Notably, in the latest RCTs evaluating total neoadjuvant therapy for stage III rectal cancer, the reported local recurrence rates remain in the range of 4%–8% [16–18].

Taken together, while some high-volume centers and selected trials report relatively low local recurrence rates, LRRC continues to be a significant clinical challenge, particularly in the management of stage III lower rectal cancer.

## The importance of surgical resection

Although conducting RCTs comparing treatment modalities for LRRC is impractical due to the nature of the disease and ethical considerations, numerous retrospective studies consistently demonstrate that R0 resection offers the best chance for long-term survival. In a Japanese retrospective multicenter study of 498 patients from 24 hospitals, the 5-year overall survival (OS) rate was 52% for patients who underwent surgical resection, compared to 44% for those treated with CIRT or proton beam therapy, 23% for those receiving chemotherapy or RT, and only 7% for patients receiving best supportive care [25]. A Swedish population-based study analyzing 426 patients similarly reported a 5-year OS rate of 33% for patients who underwent R0/1 resection, whereas the rate dropped to just 2% in those who underwent R2 resection [26]. These findings emphasize the critical role of achieving complete resection in improving prognosis.

Importantly, it is not merely the act of performing surgery that confers a survival benefit, but the ability to achieve R0 resection. A multicenter retrospective study from France involving 29 surgical centers reported 5-year OS rates of 35% for R0 resection, 12% for R1 resection, and 0% for R2 resection, with outcomes for R2 resections being comparable to those for non-resected patients [27]. Similarly, a single-center retrospective cohort study conducted in the Netherlands analyzed 447 patients with LRRC and reported a 5-year OS rate of 51% following R0 resection, 34% for R1 resection, and only 4% for patients treated with systemic chemotherapy or RT alone. Furthermore, median OS for patients undergoing R2 resection (22 months) was actually shorter than for those receiving optimal palliative CRT (29 months) [28].

While all of these studies are retrospective in nature, they collectively highlight the prognostic significance of achieving R0 resection. In contrast, R2 resection not only fails to offer survival benefit but may even be detrimental, potentially exposing patients to surgical morbidity without meaningful oncological gain. Therefore, surgical treatment for LRRC should only be considered when R0 resection is deemed technically and oncologically feasible.

## Reported R0 resection rates

Surgical resection for LRRC is technically demanding, and achieving an R0 resection remains a major challenge. The complexity of surgery varies widely depending on tumor location, anatomical involvement, and prior treatments, and surgical indications differ significantly among institutions and surgeons. As a result, the R0 resection rate is closely influenced by institutional expertise and case selection, leading to substantial inter-institutional variation (Table 2).

**Table 2:** Reported R0 resection rate for surgical resection of locally recurrent rectal cancer

Author	Year	Institution, country	Cases	Characteristics	R0 resection rate	Prognosis in R0 cases	LreRR
Moriya [31]	2004	National Cancer Center, Japan	57	PE with sacrectomy (100%)	84%	5-yr DSS: 42%	N/A
Rahbari [32]	2011	Heidelberg University, Germany	92	PE (40.2%) Sacrectomy (23.9%)	58.7%	3-yr DSS: 70%	25%
You [33]	2016	M.D. Anderson Cancer Center, USA	229	PE (35%) Bony pelvic resection (29.7%)	80.3%	5-yr OS: 50.4%	29.3%
Westberg [29]	2018	Swedish Colorectal Cancer Registry	121	–	53%	5-yr OS: 43%	20%
Iversen [34]	2018	Karolinska University, Sweden	95	Sacrectomy (26%)	77%	3-yr OS: 61%, 3-yr DFS: 41%	16%
Bird [35]	2018	Peter MacCallum Cancer Centre, Australia	98	Bony pelvic resection (34%)	66%	5-yr OS: 41.8%^a^ 5-yr PFS: 22.5%^a^	56%
Hagemans [28]	2020	Erasmus MC Cancer Institute, Netherlands	193	PE (36%)	60.1%	5-yr OS: 51%	32.1%^a^
Tanaka [36]	2020	Nagoya University, Japan	70	PE (74.3%) Bony pelvic resection (58.6%)	67.1%	5-yr OS: 64.3%	N/A
Swartjes [30]	2023	Netherlands Cancer Registry	42	–	54.8%	3-yr OS: 87%	N/A
Waller [37]	2024	Royal Prince Alfred Hospital, Australia	305	PE (100%) Sacrectomy (60.7%)	77%	5-yr OS: 45%^a^	N/A

LreRR, local re-recurrence rate; PE, pelvic exenteration; DSS, disease-specific survival; N/A, not available; OS, overall survival; DFS, disease-free survival; PFS, progression-free survival; The dashes indicate data is not shown.

^a^In all resected cases.

Population-based studies have reported R0 resection rates around 50% in real-world settings. A Swedish national population-based study analyzing 426 patients with LRRC as the first site of recurrence found that among the 121 patients who underwent curative-intent surgery, an R0 resection was achieved in 64 cases (53%) [29]. Similarly, a Dutch population-based retrospective cohort study also demonstrated the R0 resection rate for LRRC was 54.8%, although the incidence of local recurrence was very low at 6.8%, of which 55.1% were accompanied by concurrent distant metastases [30].

In contrast, high-volume specialist centers have reported significantly higher R0 rates, even in highly complicated cases. At the National Cancer Center in Japan, Moriya *et al*. reported that an 84% R0 resection rate was achieved in 57 patients undergoing pelvic exenteration (PE) with sacrectomy [31]. Other major institutions have also reported favorable outcomes: Heidelberg University (58.7%) [32], MD Anderson Cancer Center (80.3%) [33], Karolinska University Hospital (77%) [34], Peter MacCallum Cancer Centre (66%) [35], Nagoya University (67.1%) [36], Erasmus MC Cancer Institute (60.1%) [28], and Royal Prince Alfred Hospital (77%) [37].

These findings suggest that although R0 resection remains difficult to achieve in general practice, it is more frequently attained in expert centers equipped with multidisciplinary teams, advanced surgical techniques, and comprehensive perioperative care.

## Definition and assessment of R0 resection

The definition and evaluation of clear surgical margins in LRRC remain controversial and complex. Traditionally, the R classification system (R0, R1, R2) is used to describe margin status, while the CRM is widely adopted in the context of TME for primary rectal cancer. However, whether these frameworks are applicable to LRRC remains an open question. R0 resection is generally defined as the complete removal of tumor tissue with no microscopic tumor cells at the proximal, distal, or radial margins of the resected specimen. It serves as a standard metric for surgical quality in oncologic procedures [38]. In primary rectal cancer, CRM status—specifically a margin of <1 mm—is recognized as an independent predictor of local recurrence and poor prognosis, reflecting incomplete TME [1, 39].

Several studies have investigated whether CRM criteria can be extrapolated to LRRC. A retrospective study from the Netherlands reported that, in patients with LRRC, a radial margin of 0–2 mm was associated with significantly worse OS and higher rates of local re-recurrence compared to cases with margins ≥2 mm. The outcomes in patients with 0–2-mm margins were similar to those with R1 resections, highlighting the importance of securing at least a 2-mm radial margin [40]. Similarly, it has been demonstrated that both in locally advanced rectal cancer and in LRRC, a radial margin <1 mm correlated with significantly higher rates of local recurrence [41]. Importantly, the OS in these patients was also equivalent to that of R1 resection, further supporting the clinical relevance of minimum margin thresholds.

In contrast, an Australian study analyzing 210 LRRC patients who underwent PE reported that while R0 resection significantly improved survival and local control compared to R1 resection, there was no additional survival benefit from obtaining a wider radial margin. In that study, securing a radial margin of ≥0.5 mm did not result in significantly improved local control [42].

**Figure 1:**
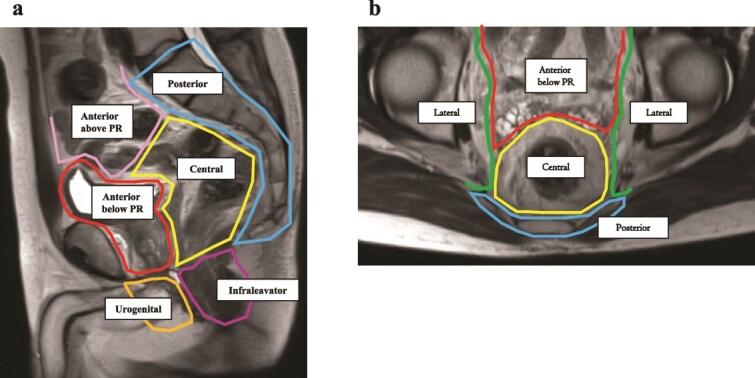
The Royal Marsden classification of locally recurrent rectal cancer. (a) Sagittal MRI view illustrating anatomical compartments. (b) Axial MRI view showing the spatial delineation of recurrence regions. *Modified from: Georgiou PA, et al. Eur J Cancer. 2013 [2] and Rokan Z, et al. BJS Open. 2021* [47]*.* PR, peritoneal reflection.

Taken together, these findings suggest that while R0 resection is essential, the optimal definition of a “clear margin” in the context of LRRC remains unresolved. Whether minimum clearances are sufficient (e.g. R0) or whether uniform criteria for threshold margins (e.g. 1 mm or 2 mm or more) should be adopted based on the concept of CRM is still under investigation (Fig. 1).

## Indication for surgery

Extended surgery for LRRC demands highly advanced surgical skills, multidisciplinary support, and meticulous perioperative management. Given the inherent risks, ensuring the safety of surgery is paramount. Regardless of the surgeon’s best intentions, serious complications or perioperative mortality are unacceptable outcomes. Therefore, any surgical indication for LRRC must begin with an objective evaluation of whether an R0 resection can be safely achieved at the treating institution, considering the technical expertise and experience of the surgical team.

There are often cases where standard PE with a permanent double stoma is not sufficient. Advanced procedures such as bony pelvic resection (involving the sacrum and ischium) and pelvic sidewall resection (involving main trunk of internal iliac vessels, internal obturator muscle, piriformis muscle, ischial spine, sciatic nerve, etc.) could be required. These surgeries are often associated with significant postoperative functional loss, such as bowel, urinary, sexual, and gait dysfunction. Moreover, even when R0 resection is technically successful, the risk of distant metastasis, especially pulmonary, and local re-recurrence remains high. Thus, a truly absolute surgical indication exists only when both the patient and their family fully understand the potential benefits and drawbacks and still strongly wish to proceed with surgery.

Unlike the relatively clear indications for surgery in primary rectal cancer, the surgical indications for LRRC are inherently more complicated, individualized, and institution dependent. However, they can be broadly divided into two main conditions.

An R0 resection is deemed technically and oncologically feasible and can be performed safely at the treating institution.

The patient and their family fully understand the risks, expected loss of function, and possible oncological outcomes, and still express a strong desire for surgical intervention.

Importantly, the first criterion is dynamic and may vary over time as surgical teams gain experience, adopt new technologies, and develop institutional capabilities.

In recent years, the boundaries of surgical indications for extended pelvic procedures have continued to expand. This evolution has been driven by advances in surgical instruments, particularly hemostatic devices, and a deeper understanding of pelvic anatomy. Procedures that were once considered relative contraindications, such as combined sciatic nerve resection or major arterial resection and reconstruction, are now selectively performed at specialized centers when patients are willing to accept the risks [43, 44].

Although the pelvis houses many organs closely tied to human dignity and quality of life (QOL), it contains few structures essential for survival. Therefore, in the absence of major bleeding or severe infection, life-threatening complications are relatively rare. When patients are willing to sacrifice function for oncological control, extended pelvic surgery for LRRC remains a powerful tool, one in which the surgeon’s skill and judgment play a decisive role.

Surgery is the cornerstone of LRRC treatment strategies. Recent advances in surgical techniques, reconstruction methods, and perioperative care have improved the safety and outcomes of complex pelvic surgery. These advances have contributed not only to improved local control and survival rates but also to improved postoperative QOL [45]. The role of the multidisciplinary team (MDT) is critical in optimizing treatment strategies for LRRC. A recent study reported that an R0 resection rate of 87.8% was achieved among patients deemed resectable by MDT consensus, suggesting the importance of collaborative decision-making and comprehensive evaluation [46].

## Classification of LRRC

When determining the surgical indication for LRRC, the anatomical site of recurrence plays a crucial role in assessing the feasibility of achieving an R0 resection. Various classification systems based on recurrence location and tumor extent have been proposed; however, no globally standardized system has yet been established. Among these, the Royal Marsden classification, developed in the UK by the Beyond TME group, provides a detailed and practical framework by dividing the pelvic cavity into seven anatomical compartments. Due to its clarity and relevance to surgical planning, this classification system is employed in the present review to discuss surgical strategies for LRRC.

Other site-based classification systems include the National Cancer Institute of Milan classification (2020) [5] and the Memorial Sloan Kettering Cancer Center (MSKCC) classification [6]. Additionally, other frameworks—such as the Wanebo classification (based on a modified TNM system) [7], and the Suzuki and Mayo Clinic systems—categorize LRRC by tumor fixation, anatomical relationships, and associated symptoms [8]. The existence of multiple classification schemes highlights the complexity of LRRC and the pressing need for a unified, widely accepted system.

The Royal Marsden classification divides the pelvis into seven compartments to aid in precise localization and surgical planning (Fig. 1) [2, 47]:

“Anterior (above peritoneal reflection)”—ureters, iliac vessels, sigmoid colon, small bowel, etc.

“Anterior (below peritoneal reflection)”—genitourinary system (seminal vesicles, prostate, uterus, vagina, ovaries, bladder/vesico-ureteric junction, proximal urethra), pubic symphysis.

“Central”—rectum/neo-rectum (intra/extra-luminal), perirectal fat or mesorectal recurrence.

“Posterior”—coccyx, pre-sacral fascia, retro-sacral space, sacrum, sciatic nerve, sciatic notch, S1 and S2 nerve roots.

“Lateral”—internal and external iliac vessels, lateral pelvic lymph nodes, piriformis muscle, internal obturator muscle.

“Infralevator”—levator ani muscles, external sphincter complex, ischio-anal fossa, ischium.

“Urogenital”—perineal body/perineal scar, vaginal introitus, distal urethra, crus penis.

Among these, “lateral compartment recurrence” presents the greatest surgical challenge. Even when technically operable, the R0 resection rate is low, and both local re-recurrence and distant metastasis rates remain high, making it the most unfavorable recurrence pattern prognostically [34, 48].

A systematic review reported that lateral recurrences account for 23.8% of LRRC cases, the second most common pattern after central recurrence (29.5%) [11]. According to MSKCC data, the R0 resection rate for lateral recurrences was just 4.3%, compared to 85.2% for axial recurrences, 33.3% for anterior recurrences, and 25% for posterior recurrences [46]. Given the technical complexity of lateral recurrences, achieving R0 resection requires a thorough understanding of three-dimensional pelvic anatomy, particularly involving the lumbosacral nerve trunk, ischial spine, piriformis muscle, sacrospinous ligament, and internal iliac vessels. Mastery of these anatomical structures is essential for improving surgical precision and minimizing complications.

In addition to lateral compartment involvement, anterior recurrences above the peritoneal reflection have also been associated with poorer OS compared to other locations [11, 47]. These results suggest that tumors located on the peritoneal reflection may correlate with disseminated recurrence patterns, highlighting the importance of accurate anatomical classification in both prognostic evaluation and treatment planning.

## Surgical treatment

As previously emphasized, achieving an R0 resection remains the cornerstone of curative surgery for LRRC. However, the technical complexity of achieving such a resection varies considerably depending on the anatomical site of recurrence. When recurrence is confined to soft tissue organs, the procedure is typically classified as “soft tissue PE,” involving resection of the bladder, prostate, uterus, and/or vagina. These cases are generally less complex, and the probability of achieving R0 resection is relatively high.

In contrast, when recurrence extends to the “bony pelvis,” “pelvic sidewall,” or “major vessels,” more advanced procedures such as “bony PE,” “pelvic sidewall exenteration,” and “vascular exenteration” are required. These surgeries demand a high level of surgical expertise and are best performed in specialized centers with multidisciplinary support, to optimize oncologic outcomes while minimizing complications and preserving QOL.

### Bony pelvic exenteration (sacrectomy)

When recurrence involves the posterior pelvis, “sacrectomy” may be necessary as part of radical treatment. The level of sacral involvement is a key factor in determining both surgical difficulty and the risk of neurological deficits. In particular, involvement of the L5/S1 nerve causes severe gait disturbances and significantly impair postoperative QOL [44].

Sacrectomy for LRRC was first reported by Wanebo *et al*. in 1981 [49]. A systematic review of 220 patients who underwent bony PE with en bloc sacrectomy reported an R0 resection rate of 78%, with a mortality rate of 2% and complication rate of 52% [50]. These data illustrate both the oncological efficacy and the morbidity risk associated with this procedure.

“High sacral amputation,” especially when involving the S1–S2 level, is technically more demanding due to proximity to the “sacroiliac joint,” and has traditionally been considered a relative contraindication owing to the risk of pelvic instability [51]. However, this view has been challenged by recent data. A multicenter study of 345 patients undergoing sacrectomy reported no significant difference in 5-year OS (53% vs. 44.1%) or cancer-specific survival (60% vs. 56.1%) between high (≥S2–S3) and low (<S2–S3) sacral involvement [52]. These results suggest that, in specialized centers, R0 resection should not be considered an absolute contraindication for high sacral lesions when technically feasible.

### Bony pelvic exenteration (pubis and ischium resection)

**Figure 2:**
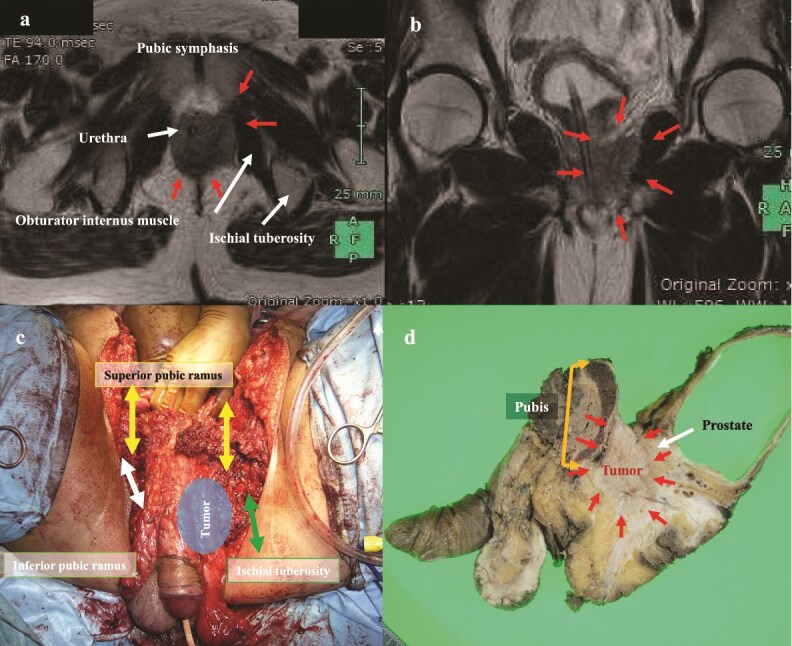
Bony pelvic exenteration (pubis and left ischium). (a, b) Preoperative MRI demonstrates that the recurrent tumor is extensively adjacent to the pubic symphysis (arrows). (c) Arrows of intraoperative view showing bilateral superior pubic rami, right inferior pubic ramus, and left ischium have been transected. (d) Resected specimen shows tumor invasion into the prostate and proximity to the pubic symphysis. Pathological examination confirmed an R0 resection. The patient recovered well, was able to walk independently without a cane, underwent liver metastasectomy 1 year later, and has achieved 5-year survival.

Although relatively uncommon, recurrence may extend to the “pubis” or “ischium,” particularly in the patterns of “anterior (below peritoneal reflection)” or “infralevator” compartments (Fig. 2). Several reports have demonstrated that these structures can be safely resected in selected patients [53, 54]. The resection of the pubic bone disrupts the continuity of the pelvic ring and causes pelvic instability. However, in cases of bone and soft tissue tumors, gait function is reported to be maintained after pubic symphysis resection, and therefore pelvic ring reconstruction is not always necessary [55]. Nevertheless, further clinical data are needed to evaluate the impact of pelvic ring resection on gait function.

### Pelvic sidewall exenteration

Surgical resection of “lateral pelvic recurrence” is technically the most demanding due to its involvement of important and complex neurovascular structures. To achieve R0 resection, it is essential to fully understand the three-dimensional anatomical structures, including the “sciatic nerve,” “internal iliac vessels,” “sacral spine,” “piriformis muscle,” “internal obturator muscle,” “sacrospinous ligament,” “sacrotuberous ligament,” and “coccygeal muscles.”

**Figure 3:**
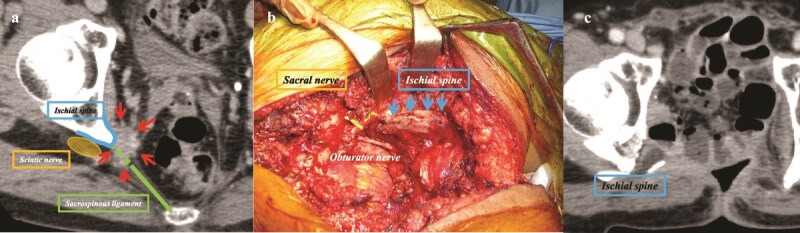
Pelvic sidewall exenteration. (a) CT imaging reveals that the recurrent tumor (arrows) invades the ischial spine and sacrospinous ligament, while sparing the sciatic nerve. (b) Intraoperative photograph following specimen removal (prone position). The sciatic nerve is visualized crossing the ischial spine and was carefully preserved by dissecting lateral to it; the ischial spine is transected. (c) Postoperative CT confirms complete resection of the recurrent tumor, ischial spine, and sacrospinous ligament as planned. The patient regained ambulatory function without the need for a cane and has survived for more than five years postoperatively.

**Figure 4:**
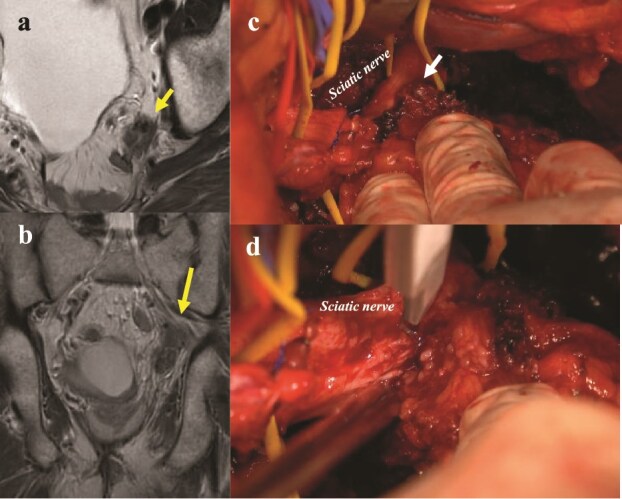
Pelvic sidewall exenteration (partial resection of the sciatic nerve). (a, b) Preoperative MRI images indicate suspected invasion of the sciatic nerve by the recurrent tumor (arrows). (c) Intraoperative photograph shows direct tumor infiltration into the sciatic nerve (arrow). (d) Partial resection of the sciatic nerve performed using a scalpel. R0 resection was achieved pathologically. Six months postoperatively, the patient is undergoing rehabilitation; walking with a cane after initial recovery with a walker.

The biggest challenge in this surgery is to preserve the “lumbar sacral trunk,” especially the “sciatic nerve,” as necessary according to the progression of the tumor, and to avoid unnecessary severe gait disorders [44] (Fig. 3). Preoperative planning is essential. A large series of 100 patients who underwent en bloc resection of the pelvic sidewall reported an R0 resection rate of 62% and a 5-year OS rate of 35%, despite a high morbidity rate of 82% and a reoperation rate of 25% [56–58]. In selected cases, “partial or complete resection of the sciatic nerve” was performed, resulting in an R0 resection rate of 65% and a 5-year local recurrence-free survival rate of 76%. Importantly, 90% of patients maintained independent mobility, suggesting that good functional outcomes are still achievable with careful planning [44] (Fig. 4).

### Vascular exenteration

When LRRC involves major vessels such as the external or common iliac arteries, vascular exenteration and reconstruction may be necessary to achieve R0 resection. These procedures carry potential risks, including graft infection, lower limb ischemia, and prolonged operative time, particularly when accompanied by bowel contamination. Despite these risks, outcomes can be favorable in selected patients.

An R0 resection rate of 58.3% was reported in cases involving the internal, external, or common iliac vessels, with 4-year OS and disease-free survival (DFS) rates of 55% and 45%, respectively [59]. Grafts used included synthetic interposition, femoral–femoral bypass, and primary anastomosis. Another report similarly noted a 38% R0 resection rate and a median DFS of over 2 years, albeit with a vascular morbidity rate of 52% [56]. These findings support the inclusion of vascular resection and reconstruction as part of extended surgical strategies for LRRC in expert centers.

### Minimally invasive surgery

Minimally invasive surgery (MIS) is widely adopted not only for rectal cancer but also for various pelvic malignant tumors such as gynecological cancer and urological cancer [20,60–64]. Its application has recently expanded to more extensive procedures such as PE. We introduced laparoscopic PE (Lap-PE) in 2013 and have reported its safety and feasibility in selected cases [65]. In Japan, Lap-PE for malignant pelvic tumors with adjacent organ invasion was approved for national health insurance in June 2024, further broadening its indications.

Lap-PE offers several potential advantages. The “shared surgical field,” a feature of MIS, enhances intraoperative communication and serves as an effective educational platform for anatomical orientation and procedural instruction. Moreover, conducting prolonged procedures within a sealed environment minimizes insensible perspiration, thereby improving fluid balance and reducing systemic stress, which may contribute to a less invasive surgical profile [66]. Despite these benefits, in cases of recurrence with extensive fibrosis or scarring, technical difficulty varies significantly between patients. Therefore, indication for Lap-PE should be carefully evaluated, based not only on the surgeon’s experience but also on objective assessment of tumor location and extent.

Robotic PE is also gaining popularity, particularly in primary cancer surgery. Nevertheless, its use remains limited to highly selected cases due to inadequate suction systems and challenges in maintaining an optimal surgical field.

A meta-analysis published in 2018 reported that only 3.8% of minimally invasive PE (MIS-PE) cases were performed for recurrent tumors, highlighting the difficulty of applying MIS to LRRC [67]. A more recent systematic review reported that MIS-PE was performed in 264 out of 2009 PEs (13.1%) [68]. This review concluded that MIS-PE is a viable alternative to open PE, offering equivalent oncological outcomes with the added benefits of reduced intraoperative blood loss and shorter postoperative hospital stays. However, these outcomes were reported from high-volume centers by expert teams, and generalization to broader settings remains uncertain.

Importantly, MIS should not be selected based on the surgeon’s preference or interest. It must never be the goal in itself. The primary aim should always be to achieve oncologically optimal resection, and the surgical approach should be selected accordingly. Nevertheless, with continued advances in technology and instrumentation, the role of MIS-PE is expected to expand in the coming years.

### Perioperative treatment

Preoperative therapy for LRRC may improve prognosis by increasing the likelihood of achieving an R0 resection. Among available strategies, preoperative RT has shown favorable results in several observational studies in terms of increasing complete tumor resection. However, the indication for RT must be tailored based on the patient’s history of pelvic irradiation.

### Preoperative treatment in radiation-naïve patients

In radiation-naïve patients, preoperative CRT has demonstrated significant efficacy. A retrospective cohort study conducted at two referral centers in the Netherlands and Sweden classified patients who underwent radical resection for LRRC into the following three groups based on irradiation history and preoperative treatment: Group A: patients who received preoperative CRT and had no previous irradiation; Group B: patients who had previous irradiation and underwent re-irradiation; and Group C: patients who had previous irradiation but did not undergo re-irradiation. The study found that the best oncologic outcomes were achieved in radiation-naïve patients who received preoperative CRT, emphasizing its value when no prior irradiation has been administered [69]. This is further supported by a systematic review of 15 studies encompassing 974 LRRC patients, which reported the highest R0 resection rate in patients receiving preoperative CRT (64%), compared to preoperative RT alone (52%) and adjuvant CRT (47%) [70]. A study from the Netherlands analyzed 193 patients with LRRC who underwent surgical resection. In 90% of cases, preoperative CRT was performed, and R0 resection was achieved in 60% of cases [28]. These data emphasize the importance of preoperative CRT to maximize the possibility of complete resection, particularly in radiation-naïve patients.

While the efficacy of preoperative CRT has been demonstrated in multiple cohort studies, the absence of prospective RCTs remains a significant limitation. To provide more robust evidence, the Japanese clinical oncology group (JCOG) 1801 trial is currently ongoing in Japan. This prospective RCT compares the outcomes of preoperative CRT followed by surgery and adjuvant chemotherapy with those of upfront surgery followed by adjuvant chemotherapy in patients with radiation-naïve LRRC [71]. The results of this trial are expected to contribute to the development of future treatment algorithms.

### Re-irradiation in previously irradiated patients

In patients with a history of pelvic irradiation, re-irradiation has emerged as a potential strategy to improve local control. Although previously considered high risk, advances in RT techniques such as intensity-modulated radiation therapy have reduced the toxicity profile of re-irradiation, making it safer and more feasible [72]. The GRECCAR 15 trial currently ongoing in France aims to compare the efficacy of a combination therapy consisting of preoperative chemotherapy followed by re-irradiation (30.6 Gy in 17 fractions) and surgery, versus chemotherapy alone followed by surgery, in previously irradiated patients [73]. The results of this trial are eagerly awaited and may provide important evidence for determining treatment strategies in this challenging subgroup.

### Role of chemotherapy

Currently, there is no definitive evidence supporting the efficacy of adjuvant chemotherapy following LRRC surgery. However, due to the high incidence of distant metastasis (particularly to the lungs), some clinicians recommend postoperative systemic chemotherapy. However, in actual clinical practice, the feasibility of adjuvant chemotherapy is often limited due to the invasiveness of LRRC surgery. As an alternative treatment, preoperative systemic chemotherapy may be considered for patients who are determined to be at high risk of distant metastasis. However, there is still a lack of evidence in this situation.

## Treatment for LRRC with distant metastases

Distant metastases are observed in up to 50% of patients with LRRC [74], presenting a major therapeutic challenge. Importantly, the presence of metastatic disease does not automatically preclude surgery. If extrapelvic metastases, such as those to the liver or lungs, are deemed technically resectable and the patient is fit for major surgery, either simultaneous or staged, may be considered. Indeed, under such challenging situations, aggressive surgical resection has been performed at specialized centers with favorable outcomes [36, 43, 44].

The PelvEx Collaborative Group has demonstrated the feasibility and safety of performing simultaneous pelvic exenteration and liver resection in patients with oligometastatic disease (defined as metastases ≤2 cm). In their cohort of 128 patients, R0 resection was achieved in 73.5%, with a 30-day mortality rate of 1.6% and a morbidity rate of 32% [75]. The 5-year OS was 54.6% in patients with R0 resection, significantly higher than the 20% observed in those with R1/2 resections.

When distant metastasis alone is present and deemed resectable, surgery is typically pursued. So, why hesitate to operate when resectable LRRC coexists? While recurrence generally reflects tumor aggressiveness, LRRC may also arise due to technical failure during initial surgery, rather than inherently poor tumor biology. This highlights the need for individualized treatment planning and multidisciplinary discussion.

When both LRRC and distant metastases require extensive surgical intervention, additional complexity arises. In such cases, a staged resection strategy is often adopted to minimize perioperative risk. However, the decision regarding which site to resect first is critical. The prolonged treatment period inherent in staged surgery may cause tumor progression or deterioration of the patient’s condition, potentially making it impossible to complete the treatment plan. Conversely, radical pelvic surgery often entails significant QOL impairments, including permanent colostomy and/or urostomy.

Given these considerations, prioritizing metastasectomy may be advantageous. By addressing distant disease first, clinicians may avoid unnecessary pelvic exenteration in patients whose condition deteriorates before the second stage of surgery can be performed [36].

## Carbon ion radiotherapy

In April 2022, CIRT was approved for coverage under Japan’s National Health Insurance system. Its indication includes unresectable local recurrence following radical resection of malignant pelvic tumors, including unresectable LRRC. In recent years, CIRT has gained increasing attention as a promising treatment option for patients for whom R0 resection is technically challenging or who decline surgery.

Historically, conventional X-ray–based RT (XRT) has yielded poor outcomes in the management of LRRC. Reported 5-year local control and OS rates have been as low as 13.1% and 23.2%, respectively [76]. In contrast, more recent evidence from a cohort of 224 LRRC patients treated with CIRT reported markedly improved outcomes, with a 5-year local control rate of 88% and a 5-year OS of 51% [77]. Further supporting its efficacy, a comparative study of re-irradiation in LRRC patients demonstrated that CIRT was superior to conventional XRT, achieving a significantly higher 3-year OS (86.4% vs. 54.5%, *P* = .005) and a significantly lower 3-year local recurrence rate (12.7% vs. 56.3%, *P* = .010) [78]. These findings suggest that CIRT may offer enhanced local control and improved long-term survival, particularly in patients who are not surgical candidates.

Currently, CIRT is available at 16 facilities worldwide including 7 in Japan. However, insurance coverage in Japan remains limited to cases where CIRT is used as a curative treatment for unresectable pelvic recurrence following radical resection of colorectal cancer. Use for prophylactic or palliative purposes, as well as for metastatic disease in other sites, is currently not approved. Given its favorable clinical outcomes, CIRT represents a valuable treatment option for inoperable or technically challenging LRRC cases. However, several important questions remain to be addressed before expanding its indications. These include determining how CIRT should be positioned relative to surgery in resectable cases, and whether it may have a future role as a preoperative treatment.

## Conclusions

The management of LRRC remains one of the most complex areas in colorectal surgery. Successful treatment requires high surgical expertise, multidisciplinary coordination, and access to specialized centers. Surgical indications vary widely among institutions, often reflecting differences in experience and resources.

While resection offers the best chance for long-term survival, attempts beyond institutional capacity can result in serious complications, reinforcing the need for careful patient selection and centralized care. Despite promising outcomes reported in retrospective studies, high-level evidence remains limited, highlighting the importance of ongoing research.

Future strategies should focus on refining surgical indications, integrating novel modalities such as CIRT and MIS, and developing tailored, evidence-based treatment pathways to improve both survival and QOL in patients with LRRC.
